# The Role of Intestinal Microbiota and Diet as Modulating Factors in the Course of Alzheimer’s and Parkinson’s Diseases

**DOI:** 10.3390/nu16020308

**Published:** 2024-01-19

**Authors:** Witold Czarnik, Piotr Fularski, Agata Gajewska, Paulina Jakubowska, Zofia Uszok, Ewelina Młynarska, Jacek Rysz, Beata Franczyk

**Affiliations:** 1 Department of Nephrocardiology, Medical University of Lodz, ul. Zeromskiego 113, 90-549 Lodz, Poland; 2 Department of Nephrology, Hypertension and Family Medicine, Medical University of Lodz, ul. Zeromskiego 113, 90-549 Lodz, Poland

**Keywords:** gut-brain axis, microbiota, neurodegenerative diseases

## Abstract

Many researchers propose manipulating microbiota to prevent and treat related diseases. The brain–gut axis is an object that remains the target of modern research, and it is not without reason that many researchers enrich it with microbiota and diet in its name. Numerous connections and mutual correlations have become the basis for seeking answers to many questions related to pathology as well as human physiology. Disorders of this homeostasis as well as dysbiosis itself accompany neurodegenerative diseases such as Alzheimer’s and Parkinson’s. Heavily dependent on external factors, modulation of the gut microbiome represents an opportunity to advance the treatment of neurodegenerative diseases. Probiotic interventions, synbiotic interventions, or fecal transplantation can undoubtedly support the biotherapeutic process. A special role is played by diet, which provides metabolites that directly affect the body and the microbiota. A holistic view of the human organism is therefore essential.

## 1. Introduction

The heterogeneity of the microbiota is observed not only between people but also within a person, depending on many factors. The entire resident ecosystem is a dynamic structure, and its composition is influenced by, among other things, diet, drugs, antibiotics, toxic substances, hormones, age, and physical activity [1]. 

In simple terms, a healthy microbiome maintains the body’s homeostasis by increasing the integrity of the intestinal barrier and metabolizing nutritional molecules from food, as well as xenobiotics and drugs. In addition, it mediates the fight against pathogens and has a real impact on the immune system, for example, by producing short-chain fatty acids (SCFAs), which are responsible for reducing systemic inflammation [2]. It can be observed that dysbiosis and the changes it entails characterize many chronic diseases such as diabetes, obesity, nonalcoholic steatohepatitis, hypertension, ulcerative colitis, and Crohn’s disease, as well as colon cancer [3]. Increasingly, the influence of the gut microbiota on the human neural system and psyche is gaining attention. Pathways may include the gut-brain axis, as well as mitochondrial, adaptive humoral immunity, and microvesicular pathways [4]. 

In two of the most common neurodegenerative diseases, Alzheimer’s and Parkinson’s, protein deposits associated with pathology are observed, there are abnormalities related to the immune system in the brain, and there are excessive amounts of highly reactive oxygen species. All of these are influenced by the microbiota [5]. Of note, the effect of nutritional support in neurodegenerative diseases is dependent on the composition and functionality of the microbiota, in view of which it is worthwhile to establish a person’s baseline enterotype. Such an intervention can be helpful not only in personalized diet selection but also in determining possible probiotic therapy [6]. The common denominator of microbiota and diet is inflammation. The essence of microbiota and diet in the course of neurodegenerative diseases will be discussed using that example. 

## 2. Mechanisms Underlying Selected Neurodegenerative Diseases

The diseases defined by neuronal decline and the ongoing deterioration of multiple regions of the nervous system are called neurodegenerative diseases. In this section, we are especially interested in the mechanisms underlying the two most frequent diseases from this group, namely the most prevalent, Alzheimer’s disease (AD), and the second in order, Parkinson’s disease (PD) [7]. 

### 2.1. Alzheimer’s Disease

The pathogenesis of AD is rooted in the formation of extracellular plaques, known as senile plaques, that mainly consist of the beta-amyloid (Aβ) peptide. This peptide is generated through the proteolytic cleavages of the amyloid precursor protein (APP), which occurs with the assistance of specific secretases [8]. However, these plaques may be present outside the cells years prior to the onset of the symptoms [9]. At least equally significant for pathogenesis is the presence of neurofibrillary tangles (NFTs) within neurons, which comprises hyperphosphorylated tau protein. Both phenomena contribute to the onset of neuronal degeneration [10]. Neuronal loss may possibly occur because of infiltration of microglia, since Aβ and NFTs are suspected of possibly exhibiting toxic properties, and both Aβ and NFTs may lead to the deterioration of synapses and elevated oxidative stress, which subsequently forces activation of the microglia and initiates local immune response, which also has neurotoxic potential [11]. However, to definitively confirm the toxicity of Aβ and NFTs, along with the described cascade of events, further research in this field is necessary, as, at this moment, it remains a subject of debate There are also other mechanisms contributing to the development of AD. Among others, these include mutations in specific genes like APP, apolipoprotein E (APOE), presenilin 1 (PSEN1), and presenilin 2 (PSEN2) but also incorporate neuronal infections caused by herpes simplex virus type 1 (HSV-1), Chlamydia species, or spirochetes. Furthermore, gut microbiota may play a crucial role, as its disruption impacts the brain–gut axis and leads to the activation of particular processes that induce occurrence or progression of AD [12]. What is more, decreased utilization of brain glucose and vascular disturbances are likely to be significant factors in influencing the course of AD [13]. As a result of the above, over time, symptoms such as memory loss, difficulties with paying bills, forgetting addresses, or other issues related to communication, social interactions, and personality might be manifested [14]. 

### 2.2. Parkinson’s Disease 

The second most prevalent neurodegenerative disease is PD. Moreover, its frequency rises with age and may impact up to 5% of individuals aged over 85 years old [15]. The fundamental role in the pathophysiology of PD is attributed to α-synuclein (α-syn), which is a neuronal protein. This protein can form aggregates that constitute the main component of inclusions within neurons, such as Lewy bodies (LBs) or Lewy neurites (LNs), which both form characteristic pathological features of PD. Aggregation of α-syn is associated with the imbalance of cellular processes and leads to loss or gain of specific toxic functions, subsequently resulting in neurotoxicity [16]. 

The aggregating process Itself has its toxic intermediate products such as proto-fibrillar and oligomeric forms. They can disturb lysosomal, proteasomal, or mitochondrial functioning; destruct the cytoskeleton and biological membranes; and modify synaptic function, which induces the loss of neurons, especially in the substantia nigra pars compacta (SNpc) region [17]. In the initial phase of PD, the pathology of LBs affects specific locations and is typically limited to the olfactory bulb. It may also impact the intermediate reticular zone or dorsal nucleus of the vagus nerve. Subsequently, in the course of PD advancement, pathology becomes evident in the midbrain, impacting the SNpc. Furthermore, increasing evidence suggests that α-syn fibril forms can be transferred in a prion-like manner between cells, which contributes to the progression of the PD [18]. SNpc contains dopaminergic neurons that undergo degeneration as the PD progresses. This results in the manifestation of nonmotor and motor symptoms. The first symptom group includes, among other symptoms, olfactory issues, mood disruptions, or dementia and may appear years before motor symptoms occur. The second group of symptoms involves bradykinesia, stiffness, rest tremors, dystonia, and difficulties in maintaining steady posture [19]. Numerous factors contribute to the pathogenesis of PD. Elements such as mutations in SNCA, PRKN, DJ-1, LRRK2, PINK1, or GBA genes or environmental components like exposure to heavy metals, contact with pesticides, farming profession, head injury, or gut microbiota alteration are considered risk factors for the occurrence of PD [20,21]. 

## 3. Gut-Brain Axis

The term “gut microbiota” refers to the microbes that inhabit the gastrointestinal tract. It has been estimated that the gut microbial species of a single individual contain over 100 trillion bacteria [22]. Fecal sample metagenomic sequencing found 3.3 × 106 non-redundant microbial genes from as many as 1500 different species [23], whereas the human genome, according to the International Human Genome Sequencing Consortium, consists of only 20,000–25,000 protein-coding genes [24]. This suggests the significance and potential impact of microbiota on human health. 

Beginning at the fetal stage, the gut microbiota gradually develops in variety and stability during the early stages of development, eventually assuming a particular composition once individuals grow up. Several factors influence that process, but the most significant ones are the delivery mode (vaginal or cesarean) [25,26], nutrition in infancy (breast milk or formula feeds) [27,28] and adulthood, and use of antibiotics or antibiotic-like compounds. The number of bacteria populating the gastrointestinal tract increases substantially from the stomach to the colon, with *Firmicutes* accounting for 60 to 80% of the total, followed by *Bacteroidetes* (20% to 40%), *Proteobacteria*, and *Actinobacteria* (5%) [22,29]. Greater diversity is generally connected with better health outcomes, yet there have been reports of higher diversity in particular illness states [30]. 

**Figure 1:**
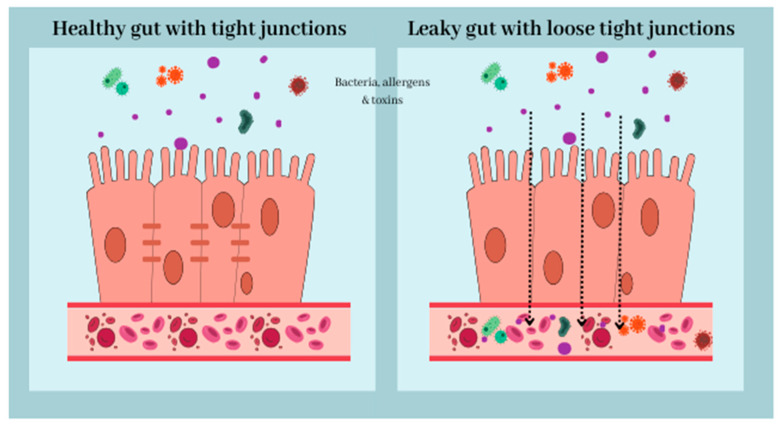
The intestinal barrier is the single, columnar epithelium cell layer. Epithelial cells are connected by desmosomes, adherence junctions, and tight junctions, which reduce paracellular permeability. Disruption of the intestinal barrier, for example, caused by dysbiosis, results in “leaky gut” syndrome. Increased gut permeability may lead to translocation of bacteria, toxins, and allergens into the bloodstream, resulting in a pro-inflammatory state.

Eubiosis, also referred to as “healthy microbiota,” is the state in the gastrointestinal tract in which the majority of potentially beneficial bacterial species coexist harmoniously. While a healthy person’s microbiome usually remains stable in a state of eubiosis, there is no doubt that food and lifestyle choices can affect the dynamics of gut microbial composition. Unhealthy habits can lead to dysbiosis, which is defined as a disturbance in the gut microbiota that disrupts intestinal homeostasis, induces inflammation, and negatively impacts intestinal permeability [31]. This relationship is demonstrated schematically in Figure 1.

Much data suggests that the intestinal microbiota also interacts bidirectionally with the central nervous system (CNS) via both direct and indirect mechanisms, which is called the gut-brain axis. Some of the most significant ways that gut microbes can interact with the central nervous system include the synthesis of neurotransmitters (acetylcholine, catecholamines, gamma-aminobutyric acid, histamine, and serotonin), immune system activity modulation, the synthesis of specific metabolites, effects on tryptophan metabolism, alterations in the microbiota’s composition, and nerve connections [32]. 

The enteric nervous system (ENS), which controls gastrointestinal function, can work independently or be regulated by the nervous system via sympathetic and parasympathetic (vagus nerve) signaling. This connection between the gut microbiota and the ENS is facilitated by the synthesis and release of a variety of chemicals, including neuropeptides and neurotransmitters, cytokines, and microbial metabolites. Furthermore, enteroendocrine cells that receive direct signals from the gut microbiota can secrete circulatory hormones that have the potential to penetrate the blood–brain barrier (BBB) and impact CNS cells [33]. 

Changes in the composition and function of the gut microbiota can result in a proinflammatory state and increased intestinal permeability. The disruption of the intestinal barrier is referred to as “leaky gut” and is primarily caused by bacterial infections, oxidative stress, alcohol, and dysbiosis. Impaired intestinal barrier function can result in the uncontrolled passage of bacterial components, toxic metabolites, and inflammatory factors, resulting in systemic inflammation [34]. Systemic inflammation triggers an elevation in the circulating levels of pro-inflammatory cytokines like interleukin (IL) 6, IL-18, and tumor necrosis factor alfa (TNF-α) [35,36,37]. Circulating inflammatory mediators impair the BBB, leading to increased BBB permeability. These inflammatory mediators not only penetrate the central nervous system but also induce the brain’s own cytokine production, which consequently promotes neuroinflammation [38].

An increasing amount of research indicates that metabolites produced by gut microbes have a significant role in the onset and progression of brain disorders. The major products of bacterial fermentation of indigestible polysaccharides and fibers are SCFAs such as acetate, propionate, and butyrate. SCFAs have been demonstrated to regulate the BBB, modify neuroplasticity, modulate microglia development, regulate neurotransmission, and promote the synthesis of serotonin [39]. Furthermore, SCFAs can influence neuroinflammation through regulating the synthesis and migration of immune cells, including neutrophils, T cells, and inflammatory cytokines [40]. 

In addition to SCFAs, a few metabolites produced by gut microbes can serve as crucial neuroactive chemicals. Some species of *Bifidobacterium* and *Lactobacillus*, for instance, can produce neurotransmitters such as gamma-aminobutyrate (GABA) and acetylcholine [41,42,43]. Serotonin can be synthesized by *Streptococcus*, *Enterococcus*, and *Escherichia*. Surprisingly, the gut is responsible for the production of more than 90% of the body’s serotonin. Other neurotransmitters, including dopamine and norepinephrine, have been shown to be produced by bacteria such as *Lactobacillus*, *Serratia*, *Bacillus*, *Morganella*, and *Klebsiella* [44]. Furthermore, some important vitamins, such as vitamin K, B2, B9, and B12, which are generated by gut microbes, can have neuroprotective effects on the CNS [45]. 

## 4. Gut Microbiota in Alzheimer’s Disease 

**Figure 2:**
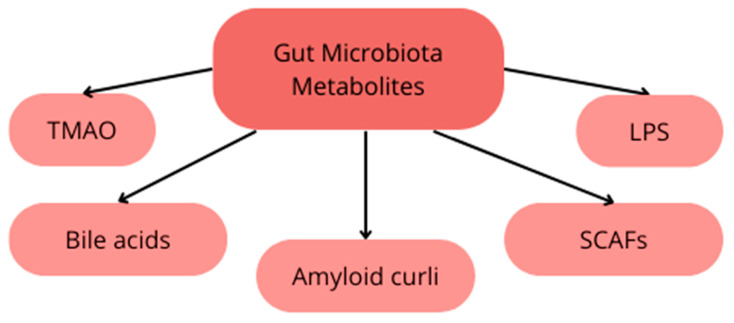
Gut Microbiota Metabolites.

The gut microbiota is believed to impact the development of neurodegenerative diseases through diverse pathways. Metabolites generated by bacteria, including trimethylamine N-oxide (TMAO), secondary bile acids (BAs), SCFAs, amyloid curli, and lipopolysaccharides (LPS), contribute to various immune and metabolic changes that may drive disease progression [46,47,48,49] as presented on Figure 2. Additionally, these metabolites could result in an increase in the permeability of the intestinal and blood–brain barriers and modulation of inflammatory responses. Furthermore, the gut microbiota influences the maturation of microglia, responsible for maintaining central nervous system (CNS) homeostasis and regulating inflammatory responses [50]. Fecal samples from patients with neurodegenerative diseases exhibit differences in species composition compared to healthy individuals, which affects the function of the microbiota. Patients with Alzheimer’s disease show an increased abundance of *Bacteroides* and a decreased abundance of *Firmicutes* and *Actinobacteria* [51]. Among patients with Parkinson’s disease, changes in the microbiota have been observed, such as an increase in abundance of *Akkermansia Muciniphila* and a decrease in abundance of *Faecalibacterium* and *Roseburia* [52]. 

TMAO is widely acknowledged as a metabolite that is associated with pro-inflammatory effects. This compound is synthesized in the liver from the precursor protein trimethylamine (TMA) through enzymatic reactions catalyzed by monooxygenases, such as FMO1 and FMO3 [53]. TMA, in turn, is produced from choline, L-carnitine, and betaine by gut bacteria, with Firmicutes and Proteobacteria playing a predominant role in this process [54]. Hence, it can be inferred that a diet rich in TMAO precursors, such as red meat, fish, and eggs, may lead to an elevated concentration of this protein in the bloodstream [55]. TMAO highly accelerates the conformational change of proteins from Aβ random coil to beta-sheet, which is essential for fiber to form [56]. In 2018, a large study with a sample size of *n* = 410 was conducted, comparing the concentrations of TMAO protein in healthy individuals (*n* = 335), individuals with AD (*n* = 40), and individuals with mild cognitive impairment (*n* = 35). The results of this study indicated that cerebrospinal fluid levels of TMAO were higher in the groups of individuals with AD and those with mild cognitive impairment compared to the group without cognitive disorders. Moreover, the study revealed a positive correlation with AD markers (phosphorylated tau and Aβ), as well as markers of neuronal degradation (total tau, neurogranin, and neurofilament light chain protein) [57]. In 2022, a study was conducted in which it was demonstrated that patients with Parkinson’s disease exhibited significantly elevated levels of TMAO pathway constituents compared to the control group [58].

Primary bile acids, synthesized by the liver, are stored in the gallbladder and under the influence of the hormone cholecystokinin, released into the gastrointestinal tract, where the majority undergoes reabsorption in the ileum. The fraction of primary bile acids that remain unabsorbed undergo numerous metabolic processes facilitated by gut bacteria, leading to their transformation into secondary bile acids [59]. Bile acids have the capacity to modulate the composition of the gut microbiota through direct cytotoxic effects on specific bacterial species [60] and the activation of inducible nitric oxide synthase (iNOS), carbonic anhydrase 12 (CAR12), and interleukin-18 (IL-18), which are associated with the inhibition of bacterial overgrowth [61]. This suggests that increased exogenic intake of bile acids may lead to disturbances in gut microbiota.

Various bile acids exhibit diverse effects on the pathophysiology of neurodegenerative diseases. For instance, it has been demonstrated that primary bile acid (BA) is efficacious in preventing the formation of AB-42 plaques [62]. Additionally, TUDCA (tauroursodeoxycholic acid) demonstrates anti-apoptotic and anti-inflammatory effects [63]. On the contrary, secondary bile acids are hypothesized to exert an impact on the development of neurodegenerative diseases. In 2018, a comprehensive study was conducted comparing the concentrations of primary and secondary bile acids in individuals with Alzheimer’s disease (AD), those with mild cognitive impairment, and healthy individuals. The results of this investigation revealed that the concentration of primary bile acids in individuals with AD is lower than in healthy individuals, while the concentration of secondary bile acids is higher in comparison to the healthy cohort [64].

In studies conducted on rodents, an elevation in the concentration of secondary bile acids (BAs) has been demonstrated to correlate with an increase in blood–brain barrier (BBB) permeability. However, as of now, there is a lack of evidence regarding whether secondary BAs exhibit a similar effect in humans [65].

Bile acids (BAs) also play a role in modulating the activity of the farnesoid X receptor, resulting in the downregulation of the expression of the cholesterol-metabolizing enzyme CYP46A. This regulatory mechanism contributes to the accumulation of cholesterol in the brain [66]. Cholesterol is essential for the proper functioning of the brain; however, when its concentration increases in cell membranes, cholesterol significantly influences the metabolisms of APP. This occurs through direct binding to APP, promoting its insertion into phospholipid monolayers, where secretase enzymes favor the amyloidogenic pathway [67].

LPS are primarily produced by Gram-negative bacteria [55]. Under the influence of increased intestinal permeability, LPS may penetrate into the central nervous system (CNS), where it plays a crucial role in modulating receptors on microglial cells. Microglial TLR2 and TLR4 are activated by LPS, amyloid, and other metabolites produced by gut microbiota. Activation of those receptors induce inflammation, cytokine production, and innate immune defense responses. More specifically, direct activation of TLR2 induces pro-inflammatory interleukin-IL-22 and interleukin-IL17A, which subsequently triggers NF-kB signaling and cyclooxygenase 2 (COX-2) [48]. On the other hand, interaction between LPS and TLR4 also induces NF-κB pathways as well as mitogen-activated protein kinase (MAPK), which lead to increased levels of IL-6, IL-12, and TNF-alfa. Interplay between TLR4 and LPS also upregulates iNOS and COX-2, which raises production of nitric oxide (NO) and prostaglandin E2 (PGE2), which lead to neuroinflammation. A prolongated state of neuroinflammation may lead to neurodegenerative changes in the CNS [68]. Proinflammatory cytokines are also responsible for upregulation of mRNA encoding for the beta-secretase protein [69]. Additionally, the production of beta-amyloid Aβ is increased under inflammatory conditions [70]. 

*Faecalibacterium* and *Roseburia*, whose presence in the gut microbiota of individuals with Parkinson’s disease is reduced, are bacteria known for their production of SCFAs. Butyrate and other SCFAs inhibit histone deacetylase, thus increasing the expression of anti-inflammatory cytokines [71]. The paragraph above has mentioned additional anti-inflammatory properties of SCFAs. The reduced production of SCFAs in patients with Parkinson’s disease may contribute to the development of neuroinflammation. *Akkermansia Muciniphila* is a bacterium that shows increased abundance in patients with Parkinson’s disease. The potential contribution of this bacterium to the development of PD is controversial, as it is also a bacterium known to produce SCFAs [72]. However, it has been demonstrated that under conditions of reduced dietary fiber intake, *Akkermansia Muciniphila* contributes to damage to the mucus layer in the intestines [73], which leads to an increase in intestinal permeability. This, in turn, exposes the intestinal neural plexus to toxins such as LPS and pesticides, ultimately resulting in the abnormal aggregation of alpha-synuclein fibrils [74].

## 5. Diet in Neurodegenerative Diseases

Despite continuous evidence and emphasis on the importance of diet in maintaining health, there is an increase in diseases of affluence. The lifestyle of many people enforces improper dietary patterns, leading to serious diseases and impaired quality of life. 

The presumed role of diet in neurodegenerative diseases is to reduce inflammation and its consequences, such as impaired neuronal function. Mitsunori Nomura et al. in a mouse model proved that beta-hydroxybutyrate, a staple in the keto diet, reduces the negative impact of LPS inflammation, and, in the long term, age-induced chronic inflammation was observed [75]. Diets that have a potential impact on the course of neurodegenerative diseases, according to PubMed searches and available results, are the ketogenic diet, MIND, and DASH [76]. 

The ketogenic diet (KD) is based on increasing the supply of fats while limiting carbohydrates. This induces the production of ketone bodies and their subsequent use as an energy substrate. The state of diet-induced ketosis is an alternative to restriction of food intake, so that the potential consequences of fasting can be reduced [77]. There are multiple pathways through which it exerts its anti-inflammatory effects: inhibiting NF-kB; decreasing levels of cytokines IL-1b and IL-6, monocyte chemoattractant protein (CCL2/MCP-1), and TNF-α; activating microglia; and increasing levels of neutrophils [78]. These substrates have been proven to reduce the amount of Aβ, positively affect mitochondrial function, and reduce the concentration of reactive oxygen species. In addition, they have a protective effect on dopaminergic neurons and prevent hyperphosphorylation of tau protein [79]. Ketone bodies have a positive effect on human cognitive processes, such as attention and memory, as well as impaired motor skills in PD [80]. Despite the ample molecular evidence of the neuroprotective nature of KD and the promising clinical effects, there is still a lack of long-term studies solidifying the validity of this diet in patients. The undoubted disadvantage of the ketogenic diet is the reduction in bacterial species diversity [81].

It is speculated that it may disturb insulin signaling and increase inflammation in the hippocampus and amyloid-β deposition, which translates into memory disorders [82].

The Mediterranean diet consists of plant products, olive oil, fish and seafood, and red wine, with a restriction of red meat. It prevents metabolic syndrome, cardiovascular disease, and cancer, as well as neurodegenerative disorders [83]. It owes its anti-inflammatory properties to numerous vitamins, carotenoids, polyphenols (e.g., resveratrol, oleuropein, and hydroxytyrosol), and polyunsaturated omega-3 fatty acids (ω3-PUFA), which also affect the state of cell membranes [84]. It helps to prevent many cognitive disorders and dementia [85,86]. Interestingly, the bioavailability of polyphenols is strongly dependent on the digestion of the substance, which depends on their structure, pH-dependent transformations, and reactions with other food ingredients. It can enter complexes with other molecules, modifying its antioxidant potential [87]. Xuejiao Qie et al. confirmed that protein has a protective character against the digestive system using the example of combining coffee with soy milk. The authors emphasized their thesis that engineers of new medical preparations should move in this direction [88].

Maria I. Maraki et al., in a sample of 1047 people, proved that the Mediterranean diet reduces the risk of prodromal Parkinson’s symptoms [89].

Another interesting prospective study includes the Mediterranean diet metabolomic score (MDMS) based on serum biomarkers of MD crucial food components. The authors concluded that higher MDMS is prospectively associated with a lower risk of age-related cognitive decline [90].

In a recent review on the effect of diet on cognition in AD, it was found that the results of the work are inconclusive due to different methodologies. However, many of the findings are promising and could form the basis for future research [91]. Another review found that the Mediterranean diet reduces the risk of AD and PD, and, in the process, the role of diet in maintaining the gut microbiota in homeostasis should be emphasized. The high concentration of polyphenols in the colon promotes the proliferation of beneficial *Lactobacillus* bacteria and the reduction of harmful ones like *Clostridium*, *Shigella*, and *Escherichia*. Attention was also drawn to the beneficial antiparasitic effects of SCFAs deriving from fiber and to the modulation of the microbiota and the growth of endotoxins. Of note, endotoxins can trigger the aggregation of Aβ and tau in AD patients and α-syn in PD patients [92]. In a study, the MeD was shown to be helpful in establishing biomarkers in the cerebrospinal fluid of AD patients, as it affects the ratio of Aβ40 and Aβ42/40, which is larger (shifts from 1:9 in healthy individuals to an average of 3:7 in AD patients) in healthy individuals and those with cognitive impairment. The researchers also noted that the dietary intervention improved cerebral blood flow and individual memory [93]. Bacteria that the Mediterranean diet promotes growth of include *Prevotella*, *Bifidobacteria*, *Eubacterium eligens, and Bacteroides*. The result is an increase in SCFA and bioactive compounds that can cross the BBB bacteria [94]. It is postulated that thanks to unsaturated fats and fiber, it reduces the risk of metabolic endotoxemia, i.e., a two-to-three-fold increase in LPS [95]. 

It is speculated that the Mediterranean-DASH Intervention for Neurodegenerative Delay (MIND) diet is more effective than the Mediterranean diet or the Dietary Approaches to Stop Hypertension (DASH) diet. The DASH diet consists of fruits, vegetables, nuts and legumes, low-fat dairy products, and whole grains. It is recommended to limit the consumption of sodium, sweetened beverages, and red and processed meat [96].

The MIND diet has vegetarian premises with a reduction in meat and saturated fats. It emphasizes the consumption of berries, green leafy vegetables, beans, nuts, whole grains, seafood, poultry, olive oil, and wine. The components of the MIND diet can be assigned the following values: 0–0.5–1. The highest scoring items, worth 1 point, are green vegetables, at least six servings consumed per week, or berries, at least two servings per week [97]. One prospective study estimated that adherence to this diet reduces the risk of AD by 53% [98]. Another study, a cross-sectional one, found that adherence to the MIND diet was positively correlated with age of onset of Parkinson’s disease, with the largest difference in the study being 17.4 years [99]. 

In general, the Western diet contains components that have been clinically associated with an unhealthy lifestyle. This dietary pattern, based on a high supply of simple sugars and unhealthy fats, which are typically found in processed food, drives metabolic syndrome, as well as dysbiosis. Consequently, it exacerbates inflammation, increases BBB permeability and amyloid accumulation in the brain, disrupts synapses, and impairs cognitive function [100]. 

## 6. Future Perspectives

Ongoing research strongly suggests an interplay between the gut microbiome and the central nervous system, influencing the development and progression of AD and PD. Potential therapeutic interventions targeting the gut-brain axis may emerge, offering novel approaches for disease management. 

There are many possible microbiota-based interventions, such as probiotics, nutritional supplements such as fish oil, or personalized dietary strategies, that could lead to new approaches for preventive measures and complementary treatments [101]. For example, synbiotics, a new fusion of food components or dietary supplements combined with probiotics and prebiotics, selectively induce the proliferation and activate the metabolism of a small group of bacteria [102]. Another studied intervention with fecal microbiota transplantation from healthy donors has indicated decreased neurodegenerative progression by reducing translocation of pro-inflammatory gut bacteria from gut to brain and consequently the neuroinflammation processes mediated by them [103]. However, studies have shown that even simple dietary changes, such as increasing fiber consumption and eating a “Mediterranean diet,” do impact disease progression [104]. Additional studies are needed to better understand the biochemical influences of these interventions, though, as well as to understand whether exposure to these interventions influence long-term disease progression or, if once the intervention is ceased, the microbiome composition reverts. 

Defining the specific mechanisms by which the microbiota impacts neurodegenerative processes may lead to innovative diagnostic tools and early intervention strategies [105]. Future studies should also consider the possible role of oral/nasal microbiota in diagnosing microbiota dysregulation, which could be taken into consideration as a future diagnostic tool, but more data are needed [106]. Other gastrointestinal factors, such as intestinal permeability and reduced intestinal motility, have been shown to be associated with higher risk of neurodegeneration [107]. 

Recognizing the uniqueness of individual microbiomes and implementing targeted improvements in individual gut microbiota health will be pivotal for advances in personalized medicine, especially against neurodegenerative diseases. 

## 7. Conclusions

In AD and PD, there is a gradual loss of neurons. Both of these pathological conditions are classified as neurodegenerative diseases. At the foundation of each of these diseases, there are complex underlying mechanisms, leading to a similar outcome, namely damage to specific neurons due to neurotoxicity. Among the many known risk factors for the occurrence of both AD and PD, disturbances in gut microbiota are receiving increasing attention in both cases. 

Numerous studies on the dynamic microbial system and its interactions with the nervous system have demonstrated that modifications to the diversity of gut microbes have an impact on neuropsychiatric health by causing modifications to the gut-brain axis pathways. The precise underlying mechanisms of most of these metabolites are unknown; however, evidence of their effects on the brain is inescapable.

In recent years, scientific research has provided evidence supporting the involvement of gut bacteria in the pathogenesis of various systemic diseases. The impact of gut microbiota on neurodegenerative diseases primarily involves alterations in intestinal barrier permeability, regulation of cytokine pathways, and harmful effects of metabolites produced by bacteria. A better understanding of the mechanisms linking gut microbiota and neurodegenerative diseases may pave the way for the development of novel and effective therapies in the future. 

Strategies related to prevention and alleviation of symptoms based on a diet with anti-inflammatory and neurotransmitter components are simple and achievable. The Mediterranean diet and the related MIND, which is a potent form of prevention and therapy, lead the way.

The current focus of research on the individual microbiome and diet can progress the medical search for promising therapy and medicine for AD and PD in the future.
